# Characterization of a Novel *TtLEA2* Gene From *Tritipyrum* and Its Transformation in Wheat to Enhance Salt Tolerance

**DOI:** 10.3389/fpls.2022.830848

**Published:** 2022-04-04

**Authors:** Zhifen Yang, Yuanhang Mu, Yiqin Wang, Fang He, Luxi Shi, Zhongming Fang, Jun Zhang, Qingqin Zhang, Guangdong Geng, Suqin Zhang

**Affiliations:** ^1^College of Agriculture, Guizhou University, Guiyang, China; ^2^Guizhou Subcenter of National Wheat Improvement Center, Guiyang, China

**Keywords:** wheat, *LEA2* from *Th. elongatum*, expression pattern, subcellular localization, interacting proteins, transformation system, salt tolerance

## Abstract

Late embryogenesis-abundant (LEA) proteins are critical in helping plants cope with salt stress. “Y1805” is a salt-tolerant *Tritipyrum.* We identified a “Y1805”-specific LEA gene that was expressed highly and sensitively under salt stress using transcriptome analysis. The novel group 2 LEA gene (*TtLEA2-1*) was cloned from “Y1805.” *TtLEA2-1* contained a 453 bp open reading frame encoding an 151-amino-acid protein that showed maximum sequence identity (77.00%) with *Thinopyrum elongatum* by phylogenetic analysis. It was mainly found to be expressed highly in the roots by qRT-PCR analysis and was located in the whole cell. Forty-eight candidate proteins believed to interact with TtLEA2-1 were confirmed by yeast two-hybrid analysis. These interacting proteins were mainly enriched in “environmental information processing,” “glycan biosynthesis and metabolism,” and “carbohydrate metabolism.” Protein-protein interaction analysis indicated that the translation-related 40S ribosomal protein SA was the central node. An efficient wheat transformation system has been established. A coleoptile length of 2 cm, an *Agrobacteria* cell density of 0.55–0.60 OD_600_, and 15 KPa vacuum pressure were ideal for common wheat transformation, with an efficiency of up to 43.15%. Overexpression of *TaLEA2-1* in wheat “1718” led to greater height, stronger roots, and higher catalase activity than in wild type seedlings. *TaLEA2-1* conferred enhanced salt tolerance in transgenic wheat and may be a valuable gene for genetic modification in crops.

## Introduction

Common wheat (*Triticum aestivum* L.) is an important grain crop worldwide and has more influence on global food security than any other crop (Wingen et al., 2017). Salt stress is one of the major abiotic stresses affecting different crops and their yields worldwide (Bhagi et al., 2013). Wheat productivity is severely affected by soil salinity. Exposure of plants to high salt stress stimulates the expression of genes encoding protective proteins such as late embryogenesis-abundant (LEA) proteins, osmotin, pathogenesis-related proteins, and ion transporters (Filho et al., 2003; Rorat, 2006; Jyothsnakumari et al., 2009). LEA proteins have been closely linked to abiotic stress tolerance, such as high salinity stress, drought, and low temperature in many studies. The *LEA* gene family is an important group of functional proteins that reduce cell damage and protect cells under abiotic stress (Hirayama and Shinozaki, 2010; Debnath et al., 2011). LEA proteins are highly hydrophilic and intrinsically unstructured in the hydrated state but partially fold into α-helical structures under dehydration (Hand et al., 2011; Thalhammer and Hincha, 2013). This feature allows them to function as chaperones, preventing protein aggregation during abiotic stress (Hanin et al., 2011; Cuevas-Velazquez et al., 2014). In addition, LEA proteins contribute to the stabilization of membranes, binding of calcium and other metal ions, and interactions between DNA and RNA (Tolleter et al., 2010; Rahman et al., 2011; Battaglia and Covarrubias, 2013; Rosales et al., 2014). LEA proteins have various protective activities against enzymes (Amara et al., 2014; Furuki and Sakurai, 2016).

The expression of *LEA* may be induced by salinity and drought stress and increase tolerance to salinity and drought stress in many kinds of plants. Overexpression of *OsLEA5* increased drought tolerance in rice (Huang et al., 2018). Transgenic sweet potato non-embryogenic calli that overexpressed *IbLEA14* showed enhanced tolerance to salt and drought stress, while RNAi calli exhibited increased stress sensitivity (Park et al., 2011). The group 5 LEA protein, ZmLEA5C, enhances tolerance to osmotic stress in transgenic tobacco (Liu et al., 2014). Despite the universal importance of common wheat, its transformation has lagged behind that of other crop species in the past few decades due to its complex polyploid genome, genotype dependence, and low regeneration following genetic transformation (Bhalla, 2006; Risacher et al., 2009; Wang et al., 2018; Hayta et al., 2019). Transgenic approaches are required for gene functional studies, crop improvement, and the delivery of components for new breeding technologies such as genome editing (Borrill et al., 2019).

The E genome species (e.g., halophile wheatgrass *Thinopyrum elongatum*) of the *Triticeae* possess salt-tolerant properties, making them invaluable sources for genetic variation and the improvement of wheat crops (Margiotta et al., 2020). *Tritipyrum* derived from the wide crosses of *Triticum* and *Thinopyrum* displays salt tolerance (Yuan and Tomita, 2015). The *LEA* genes have been reported in various plants such as bread wheat (Liu et al., 2019), *Oryza sativa* (Hu et al., 2016), peanut (Qiao et al., 2021), pepper (Luo et al., 2019), grapevine (Xu et al., 2020), and poplar (Cheng et al., 2021). However, the characterization and transformation of *LEA* genes from salt-tolerant *Tritipyrum* remain unknown. Therefore, in the present study, we cloned a novel *LEA* gene from *Tritipyrum* to understand its sequence characterization, evolutionary relationships, expression patterns, and interacting proteins under salt stress. Moreover, we established an efficient wheat transformation system (coleoptile meristem infiltration). This study provides further insight into the salt-tolerant mechanism of *LEA* genes and the breeding of salt-tolerant wheat.

## Materials and Methods

### Plant Materials

Salt-tolerant “Y1805” is a stable progeny from a wide cross between *Triticum aestivum* and *Th. elongatum*. “Y1805” not only has A, B, and D chromosomes from wheat parents but also contains a set of alien chromosomes that originated from the E genome of *Th. elongatum*. Salt-tolerant *Tritipyrum* (“Y1805”) and salt-sensitive *Triticum aestivum* (“Chinese Spring,” “88,” “811,” and “1718”) were used in this experiment.

### Plant Growth Conditions and Stress Treatments

The seeds of “Y1805” and “Chinese Spring” (CS) were germinated in a growth chamber (relative humidity of 75% and a 20/15°C light/dark photocycle). The seedlings were sown on a floater board in 1/2 Hoagland’s solution with a 16/8 h light/dark cycle, irradiance of 400 μmol m^–2^s^–1^, and the same temperature and humidity as in the germination chamber. The culture solution was refreshed every 3 days. On the 14th day (two-leaf stage), salt stress treatments (1/2 Hoagland’s solution supplemented with 250 mM NaCl) were started. The first wheat root, stem, and leaf samples of uniform size were selected at 5 h after exposure to salt stress. The materials were recovered (in 1/2 Hoagland’s solution without NaCl) after 24 h of salt stress. The second samplings were performed at 1 h after recovery. Normal (1/2 Hoagland’s solution without NaCl) cultured materials, CK1 and CK2, were used as parallel controls, respectively. All tissue samples were immediately frozen in liquid nitrogen after sampling and stored at −80°C for the transcriptomic and qRT-PCR analysis, and gene cloning. Three biological replications were used, and at least 10 seedlings were mixed per replicate.

### RNA Extraction and Transcriptome Analysis

Total RNA was extracted from the roots at the salt stress and recovery stages using TRIzol reagent (Thermo Fisher Scientific, Waltham, MA, United States) following the manufacturer’s protocol and then treated with RNase-free DNase I (Takara, Dalian, China) for 30 min. The total RNA quantity and quality were identified by both 1.0% agarose gel and a NanoDrop 1000 spectrophotometer. A total of 20 μg RNA was used for cDNA library construction and transcriptome sequencing (BGISEQ-500) at Beijing Genomics Institute (Shenzhen, China). After data filtering, clean reads were obtained and then compared with the reference genomes [common wheat “CS,” AABBDD^1^ and *Th. elongatum*, EE^2^] by using HISAT2 (version 2.1.0) software. Differentially expressed genes (DEGs) were selected according to the method of Peng et al. (2022). In brief, fragments per kilobase of exon model per million mapped fragment (FPKM) values were calculated using RSEM (version 1.2.8), and the level of gene expression was quantified (Bates et al., 1973). The FPKM method was then used to detect DEGs among the treatment and control samples (Trapnell et al., 2010). Finally, genes potentially regulated by treatment were identified using a false discovery rate (FDR) threshold < 0.01, *p*-value < 0.001, and absolute log_2_fold change value (| log_2_FC|) > 1 between the three salt-treated and three salt-free samples using DESeq software (Anders and Huber, 2010). The Phyper function in the R package was used for enrichment analysis of gene ontology (GO).

### RNA Reverse Transcription, Late Embryogenesis-Abundant Gene Amplification, and Plasmid Construction

A PrimeScript RT kit (Takara) was used to reverse transcribe RNA into cDNA. The full-length coding sequence of *TtLEA2-1* was amplified from “Y1805” cDNA using primers with a *Bsa*I restriction site at the 5′ and 3′ ends of the amplified fragment. The primers used for amplification are provided in Supplementary Table 1. The amplified fragment was digested with Sac/*Spe*I and *Bam*HI/*Kpn*I, and inserted into the pTCK303 vector using T4-DNA ligase (Takara) as described by the manufacturer’s protocol. This vector has been modified to contain the green fluorescence protein (GFP) gene. The inserted sequence was driven by a cauliflower mosaic virus (CaMV) 35S promoter.

### Sequence Alignments and Phylogenetic Analysis of *TtLEA2-1* Gene

Multiple alignment of the *LEA* gene sequences was performed using DNAMAN with the complete alignment method. Displaying complete base sequence and coloring 100% homologous bases were selected as the parameters. Online tools Expasy^3^ for hydropathy prediction, and NetPhos 2.0^4^ and CPHmodels 3.2^5^ for phosphorylation site analysis were adopted.

TtLEA2-1 proteins were identified from wheat protein sequence data using BLAST HMM profiles^6^ for LEA_1 (PF03760), LEA_2 (PF03168), LEA_3 (PF03242), LEA_4 (PF02987), LEA_5 (PF00477), LEA_6 (PF10714), seed maturation protein (SMP, PF04927), and dehydrin (DHN, PF00257) (Liu et al., 2019). A total of 57 DNA sequences encoding LEAs were retrieved from the Phytozome^7^ and Ensembl^8^ servers (accession numbers in Supplementary Table 2). A phylogenetic tree was created using MEGA 7 (Kumar et al., 2016) with the maximum-likelihood method with 1000 bootstraps.

### Homology Modeling and Molecular Simulation of TtLEA2-1 Protein

MODELLER9.22^9^ was adopted for homology modeling of TtLEA2-1 protein (Eswar et al., 2006). The X-ray crystal structure of a putative LEA protein At2g46140.1 (PDB ID: IYYC) was used as template for this procedure. The predicted model was analyzed using SAVES^10^. GROMACS software^11^ was used for calculating root mean square deviation (RMSD) and the potential energy value of the model protein (Hess et al., 2008). Ramachandran plots were analyzed using Rampage server^12^ (Lovell et al., 2003).

### Expression Pattern Analysis by Quantitative RT-PCR

Total RNA from the different tissues or stages were reverse-transcribed using Power SYBR Green PCR Master Mix (Applied Biosystems, Foster City, CA, United States). qRT-PCR amplification was performed on an ABI StepOne Real-Time PCR System. The relative expression levels were calculated using the 2^–ΔΔCt^ method, with three biological replications and three technical replications (Choa et al., 2015), and β*-actin* and *18S RNA* were used as the internal controls.

### Subcellular Localization of TtLEA2-1

The construct containing TtLEA2-1 was transiently transformed into mesophyll protoplasts prepared from the leaves of 4-week-old *Oryza sativa* with a polyethylene glycol-mediated protocol (Yoo et al., 2007). The transformed mesophyll protoplasts were incubated at 22°C for 16 h in the dark. GFP fluorescence was observed in transformed protoplasts using a confocal laser-scanning microscope (FV1000 Olympus Corp., Tokyo, Japan).

### Yeast Two-Hybrid Screen of TtLEA2-1 Interacting Proteins

An Ultrapure RNA Kit (CWBIO, China) was used to extract total RNA from “Y1805” roots subjected to 5 h of salt stress following the manufacturer’s instructions. mRNA was isolated using a NucleoTrap mRNA kit (Clontech, Carlsbad, CA, United States). Double-strand cDNA was synthesized using a SMART cDNA Library Construction Kit (Clontech). The double-strand cDNA was normalized using the Trimmer-Direct cDNA normalization kit (Evrogen, Moscow, Russia). Biotin-DSN-attB2/attB1 primers were added to synthesize a cDNA library. BP Clonase II Mixp (Thermo Fisher Scientific) was added to recombine pDONR222 (ZYbscience, Shanghai, China) with cDNA, which was then electro-transformed into *Escherichia coli* DH5α (Collaborative Innovation Center for the Prevention and Control of Infectious Diseases in the Western Region, Xi’an, China). A PureLink HiPure Plasmid Filter Midiprep Kit (Thermo Fisher Scientific) was employed to extract library plasmids. The library plasmids and pGADT7 (Takara) were co-transformed to *E. coli* DH5α (Collaborative Innovation Center for the Prevention and Control of Infectious Diseases in the Western Region). The bacterial stock solution was diluted 1000-fold to identify the capacity of the cDNA library. The full-length coding sequence of *TtLEA2-1* was inserted into the bait vector pGBKT7 (Takara). The recombinant construct was introduced into the yeast strain Y2HGold using the polyethylene glycol/LiAc method and tested for autoactivation and toxicity. SD/-Leu/-Trp (DDO) plates (Coolaber, Beijing, China) and SD/-Ade/-His/-Leu/-Trp (QDO) plates (Coolaber) were used to screen monoclonal colonies (> 2 mm), and selected colonies were placed in DDO liquid medium with shaking during the logarithmic growth phase (29°C, 200 rpm, 20 h) and then plated on QDO medium. Then positive clones were selected on DDO and QDO (Coolaber) media. The positive clones sequencing results were analyzed via the basic local alignment search tool (BLAST).

### Switch Back Prey Plasmid and Confirmation of Positive Interactions in Yeast

The selected positive clones were extracted using a yeast plasmid extraction kit (Solarbio, Beijing, China), and then transformed into *E. coli DH5*α (Collaborative Innovation Center for the Prevention and Control of Infectious Diseases in the Western Region). After culturing, the plasmid was extracted with a plasmid extraction kit (Tiangen, Beijing, China), and the plasmid and pGBKT7- TtLEA2-1 were co-transformed into yeast Y2HGold cells. The positive bacteria were screened by coating DDO, QDO, and QDO/X auxotrophic plates. The GO and Kyoto Encyclopedia of Genes and Genomes (KEGG) enrichment analyses, and protein-protein interaction (PPI) analysis were performed for these interacting proteins.

### Genetic Transformation and Phenotype Analysis

The vector with target genes was transferred into *Agrobacterium tumefaciens* strain EHA105 (Takara). *Agrobacterium* was cultivated in Luria Broth medium with 20 mg/L rifampicin, 50 mg/L kanamycin, 1 mL/L acetosyringone (AS), and 200 uL/L SILWET1-77 (pH 7.00). *Agrobacterium* containing the target gene was introduced into 120 wheat coleoptiles by *Agrobacterium*-mediated transformation under optimized vacuum pressure (coleoptile transformation). Briefly, the tip of coleoptiles was cut to expose the meristem, soaked in *Agrobacterium* inoculum in a bell jar under vacuum, and a vacuum was drawn for 5 min infiltration. Several key factors (coleoptile length, *Agrobacterium* cell density (OD_600nm_), and vacuum pressure) were screened based on gradient tests. Inoculated coleoptiles were covered with a plastic bag to maintain high humidity and placed in the dark for 3 days. These coleoptiles were then transferred onto a 24-hole tray with peat substrate.

For the selection of transformants, seedlings of T_0_ transgenic wheat containing the GFP reporter gene were detected using a LUYOR-3415RG hand-held lamp (LUYOR Corporation, Shanghai, China), and planted in a field. Their leaves were sampled at the seedling stage for further PCR identification of transgenic plants. Putative transformants were tested by multiplex PCR amplification of genomic DNA using the specific primers for the CaMV *35S* promoter and *GFP* gene, and the primers for housekeeping *18S* gene (Supplementary Table 1). PCR products were separated on 1% (w/v) agarose gel. Homozygous T3 transgenic wheat lines were selected following assays for growth (root length and seedling height), plant water content, and catalase (CAT) activity. Three biological replications were performed in this experiment.

### Plant Water Content and Catalase Activity Assay in Transgenic Wheat

Samples were collected at 0 h (CK), 5 h (T1 stage), and 24 h (T2 stage) after salt stress, and at 1 h (R1 stage) after recovery for assaying growth, plant water content, and CAT activity. Plant dry weight was measured after plants were oven-dried at 90°C for 48 h. Plant water content was calculated as (plant fresh weight - plant dry weight)/plant fresh weight × 100. An assay kit (Cas no.: BC0205, Solarbio, Beijing, China) for CAT activity measurement was applied in accordance with the manufacturer’s instructions.

### Statistical Analyses

Statistical software (SPSS 20.00, IBM Inc., Armonk, NY, United States) and graphics software (Origin 2017, OriginLab Inc., Northampton, MA, United States) were used for the data analysis and figure construction, respectively. Duncan’s multiple range test was performed to determine significant differences between means at a significance level of p < 0.05 after displaying a significant effect during an ANOVA.

## Results

### Transcriptomic Analysis of Late Embryogenesis-Abundant Expression Level Under Salt Stress and Recovery Conditions in Two Wheat Varieties

We conducted a genome-wide transcriptome analysis to determine the *Tritipyrum* genes that affected the salt stress response. LEA-related genes were found to take part in the response to salt stress using transcriptome analysis in salt-tolerant *Tritipyrum* “Y1805.” BLAST analysis indicated that *Tel3E01G270600* should be an *LEA* gene with an LEA-2 domain. Therefore, *Tel3E01G270600* may be an LEA-2 protein. The relative expression level (log_2_FC = 7.93) of *Tel3E01G270600* was significantly higher than salt-sensitive wheat CS (log_2_FC = 0) under salt stress (Table 1). However, this value returned to zero rapidly after recovery in “Y1805.” The *Tel3E01G270600* gene was annotated and assigned to oxidoreductase activity (GO:0016702) and oxylipin biosynthetic process (GO:0031408) by GO analysis. Oxidoreductase activity is closely related to ROS scavenging, and oxylipins regulate growth and responses to environmental stimuli of organisms, which plays a key role in dehydration and osmotic stress. Therefore, “Y1805”-specific *Tel3E01G270600* might be an important gene involved in the salt stress response.

**TABLE 1:** Relative expression level (log_2_fold change) of *Tel3E01G270600* under salt stress and recovery conditions in two wheat varieties.

Treatment	“Y1805”	“Chinese Spring”
Salt stress	7.93	0
Recovery	0	0

### *TtLEA2-1* Cloning and Sequence Analysis

**FIGURE 1:**
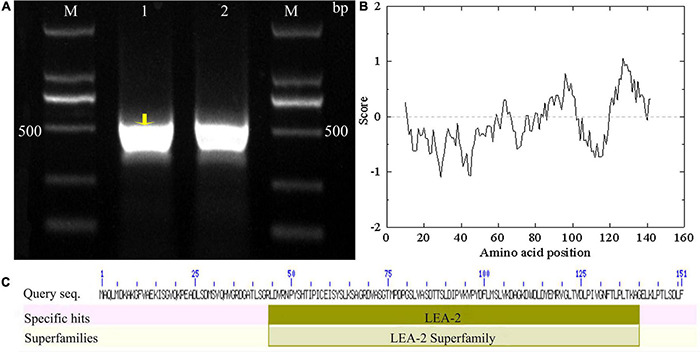
Molecular identification of *TtLEA2-1* from *Tritipyrum* “Y1805”. **(A)** Amplified bands with “Y1805” cDNA as a template; M, 2000 bp DNA marker; 1–2: “Y1805” cDNA. **(B)** Hydropathy analysis of TtLEA2-1 protein. **(C)** TtLEA2-1 protein domain.

Using *Tel3E01G270600*-specific primers, a 453 bp cDNA fragment corresponding to *Tel3E01G270600* was amplified and cloned from *Tritipyrum* “Y1805” by PCR (Figure 1A) and named *TtLEA2-1*. The *TtLEA2-1* sequence had 97.37% identity to *Tel3E01G270600*, with only 11 bp nucleotides changes between them (Supplementary Figure 1). Therefore, TtLEA2-1 was similar to *Tel3E01G270600* according to their cDNA sequences.

According to bioinformatics analysis, TtLEA2-1 encoded 151 amino acids (aa). The TtLEA2-1 protein contained seven serine, four threonine, and two tyrosine residues, which could be protein kinase phosphorylation sites. Hydropathy plots predicted that TtLEA2-1 was predominantly hydrophilic, indicating it could play a key role in plant salt-stress tolerance (Figure 1B). The TtLEA2-1 protein had an LEA_2 conserved domain at 44–40 aa, and the region was located at the C-terminal (Figure 1C), which might participate in the formation of a homotype or heterodimer and transcription regulation.

**FIGURE 2:**
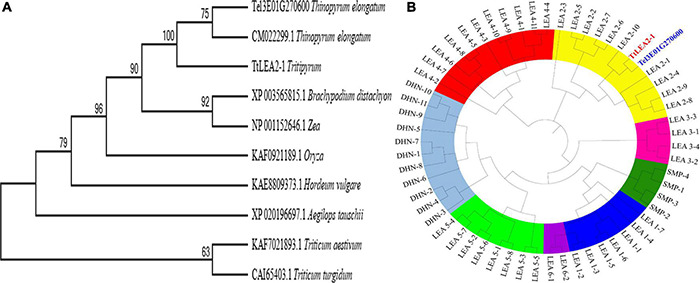
Phylogenetic relationships of *LEA* genes. **(A)** Phylogenetic tree of LEA proteins in various plant species with LEA homologs. **(B)** Phylogenetic tree of various *LEA* genes in wheat, *Tritipyrum*, and *Th. elongatum*. The Maximum Likelihood (ML) tree was generated using MEGA7 with 1000 bootstrap replicates. Bootstrap values are indicated at the branches. Wheat LEA groups are distinguished by color. Blue, yellow, rose red, red, light green, purple, green, and gray represent LEA-1, LEA-2, LEA-3, LEA-4, LEA-5, LEA-6, SMP, and DHN gene families, respectively. TtLEA2-1 and Tel3E01G270600 are shown in red and blue, respectively.

A phylogenetic tree based on aa sequences of different species showed that TtLEA2-1 displayed maximum homology with *Th. elongatum* (CM022299.1, Figure 2A). Their aa sequence identity was 77.00%, and their conserved domain was very similar. The genetic distance of TtLEA2-1 with *Th. elongatum* was closer than *Triticum* crops. The phylogenetic tree of wheat LEA proteins was also constructed, and both TtLEA2-1 and Tel3E01G270600 were clustered together into group 2 LEA proteins (Figure 2B). TtLEA2-1 showed higher similarity with Tel3E01G270600 (*Th. elongatum*) and LEA2-10 (common wheat) than with other LEA proteins, and these were clustered in the same branch. Therefore, it was speculated that TtLEA2-1 could have a similarity and close genetic relationship with group 2 LEA protein of *Th. elongatum* in evolution and function.

### Homology Modeling and Molecular Simulation of TtLEA2-1 Protein

**FIGURE 3:**
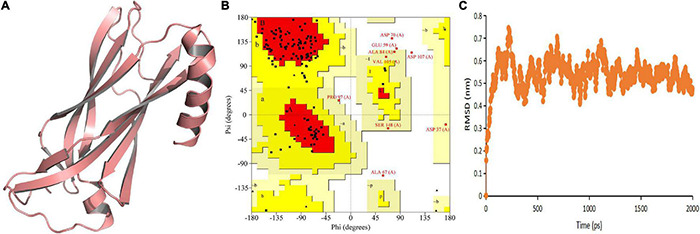
Homology modeling and molecular simulation of TtLEA2-1 protein. **(A)** Homology modeling of TtLEA2-1 protein using MODELLER9.22. **(B)** Ramachandran plot analysis. A, B, and L regions: most favored residues; a, b, l, and p regions: additional allowed residues; ∼a, ∼b, ∼l, and ∼p regions: generously allowed residues. **(C)** Molecular dynamics simulation. Backbone of root mean squared deviation (RMSD) plotted versus time in picoseconds (ps).

The predicted model indicated that both N-terminal and C-terminal regions of TtLEA2-1 have an α + β-folding chain (consisting of one α-helix and some β-strands that run anti-parallel to each other) (Figure 3A). The models showed compatibility of 84.77% with At2g46140.1, indicating good quality models. Ramachandran plot validation of the protein signified its aptness since no residues were present in disallowed regions (Figure 3B). The RMSD of TtLEA2-1 protein reached a peak (0.7434 nm) at 218 picoseconds (ps) (Figure 3C). The RMSD curve arrived at equilibrium after 250 ps, with fluctuation keeping it in the range of 0.40–0.70 nm. These results demonstrated that TtLEA2-1 had reached the equilibrium state and its structure was stable.

### Expression Patterns of *TtLEA2-1* Gene in “Y1805”

**FIGURE 4:**
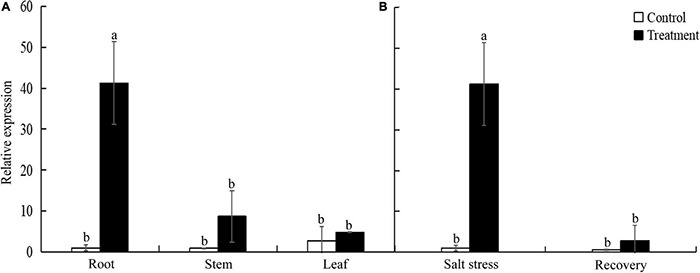
Expression levels of *TtLEA2-1* in “Y1805” measured by qRT-PCR analysis. **(A)** Relative expression levels of *TtLEA2-1* in roots, stems, and leaves under salt stress. **(B)** Relative expression levels of *TtLEA2-1* in roots under salt stress and recovery conditions. Bars indicate means with SDs (*n* = 3). Values with different letters are significantly different at *p* < 0.05.

To investigate the spatial and temporal expression pattern of *TtLEA2-1*, we analyzed the expression levels of *TtLEA2-1* in roots under salt stress and recovery conditions and in various tissues using qRT-PCR analysis. The relative expression level of the *TtLEA2-1* gene was the highest under salt stress in the roots of “Y1805,” next was stems, and then leaves (Figure 4A). Its expression level in the roots was 4.76-fold and 8.55-fold higher than those of stems and leaves, respectively. In addition, the *TtLEA2-1* expression level was significantly (40.36-fold) higher than the control under salt stress in the roots. However, it dropped rapidly to the control level after recovery (Figure 4B). These results were consistent with the above transcriptome data, indicating that “Y1805”-specific *TtLEA2-1* expressed highly and sensitively in the roots under high salinity to adapt to salt stress.

### Subcellular Localization of TtLEA2-1

**FIGURE 5:**
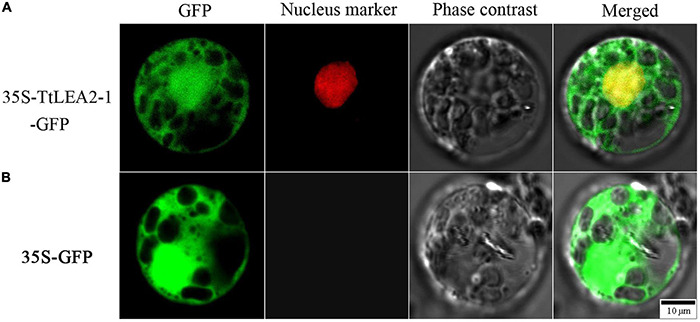
Subcellular localization of the TtLEA2-1 protein in rice protoplasts. **(A)** Fusion protein construct 35S-TtLEA2-1-GFP was introduced into rice protoplasts. **(B)** The vector control 35S-GFP was introduced into rice protoplasts. For protoplast transformation, the nuclear marker D53-mCherry was co-transformed into protoplasts and green fluorescence protein (GFP) was detected at 16 h using a confocal laser-scanning microscope. GFP fluorescence (green), nuclear fluorescence (red), merged images (green and red), and bright-field phase-contrast images are shown. Scale bar = 10 μm.

To determine the subcellular localization of TtLEA2-1, a fusion protein transiently expressing 35S-TtLEA2-1-GFP in *Oryza sativa* mesophyll protoplasts was produced. It was found that the fluorescence emitted by the fusion protein was localized to the nucleus and overlapped with the red nuclear mCherry signal (Figure 5). In addition, GFP signals were distributed throughout the cell, implying that TtLEA2-1 distributes in the whole cell. The results showed that TtLEA2-1 might contribute to transcription regulation or the protection of heredity substances and cellular components.

### Interacting Protein Analysis of TtLEA2-1

**FIGURE 6:**
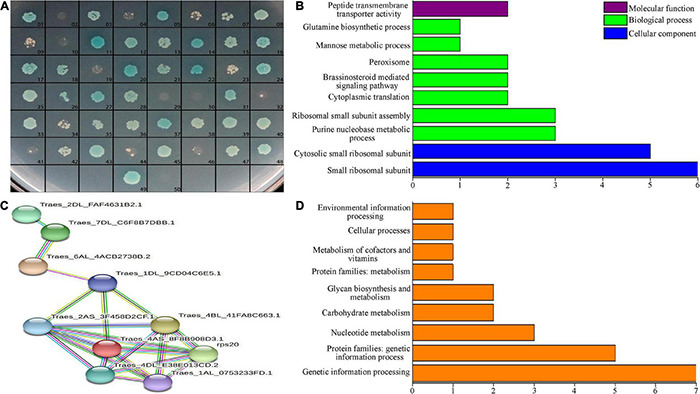
Interacting-protein analysis of TtLEA2-1. **(A)** Reversion verification of proteins with the potential to interact with TtLEA2-1. 49, positive control (yeast cells transformed with pGBKT7-TtLEA2-1), 50, negative control. Blue plaques indicate proteins with strong interaction. **(B)** Main GO terms, **(C)** PPI analysis, and **(D)** KEGG pathways of the TtLEA2-1 potential interacting protein genes.

We used the Y2H method to screen for proteins that interacted with TtLEA2-1 in the cDNA library. The pGBKT7-TtLEA2-1 vector was transformed into yeast Y2HGold cells for Y2H screening, and single clones that grew well on the DDO plate were chosen. There were white plaques on the DDO plate and sterile plaques on the QDO plate, indicating that TtLEA2-1 was a non-toxic and non-self-activating protein (Supplementary Figure 2). The pGBKT7-TtLEA2-1 and pGADT7-cDNA plasmids were co-transferred into yeast cells and cultured. After screening for defective media, preliminary screening on DDO plates, and then database comparison with QDO selection and sequencing, we found that 48 proteins interacted with TtLEA2-1. The reversion verification test found that after screening by DDO, QDO, and QDO/X auxotrophic plates, all 48 proteins contained interaction signals with TtLEA2-1 (Figure 6A).

Overall, 48 clones represented by blue plaques were confirmed by PCR and sequencing. Sequencing data and statistical results of proteins with the potential to interact with TtLEA2-1 are shown in Supplementary Table 3. By BLAST comparison, GO annotation, and PPI analysis of TtLEA2-1 interacting proteins, PPI occurred in 11 proteins (Figures 6B,C). Most of these were ribosomal proteins and were enriched in the process of protein synthesis. Among these proteins, the translation-related 40S ribosomal protein SA was the central node, suggesting that it might play a key role in the salt-stress response of “Y1805” (Figures 6B,C).

Twenty proteins could be annotated to known functional groups using KEGG pathway analysis. Half (10) of these proteins were enriched in “genetic information processing,” three in “glycan biosynthesis and metabolism,” three in “nuclear metabolism,” two in “carbohydrate metabolism,” one in “cellular process,” and one in “environmental information processing” (Figure 6D).

### Coleoptile Transformation in Common Wheat

**FIGURE 7:**
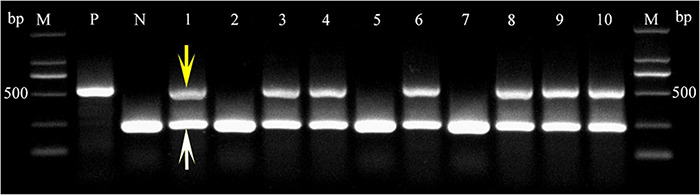
PCR confirmation of transgenic plants harboring the *TtLEA2-1* gene. PCR detection of the *TtLEA2-1* gene in the genomic DNA of putative transgenic T_0_ plant leaves. The 453 bp fragment of the amplified *TtLEA2-1* gene is indicated by a yellow arrow. A white arrow shows the band of the amplified housekeeping *18S* gene. M, 2,000 bp DNA marker; lanes 1, 3, 4, 6, 8–10, transgenic plants; lanes 2, 5, and 7, non-transformed plants; P, positive control (*TtLEA2-1* recombinant plasmid); N, negative control (wild type DNA).

To investigate *TaLEA2-1* function, overexpressed *TaLEA2-1* was transformed in common wheat plants. The coleoptile tips of common wheat were cut to expose the meristem, and infected with *Agrobacteria* containing the *TaLEA2-1* gene. Transgenic positive plants were screened firstly by a LUYOR-3415RG hand-held lamp. Then, they were confirmed by multiplex PCR using *TaLEA2-1* specific primers and housekeeping *18S* gene primers. Two bands were amplified in lanes 1, 3, 4, 6, 8, 9, and 10, indicating that DNA was from transgenic plants (Figure 7). A single band from the housekeeping *18S* gene was found in lanes 2, 5, and 7, indicating that DNA was from non-transformed plants.

**FIGURE 8:**
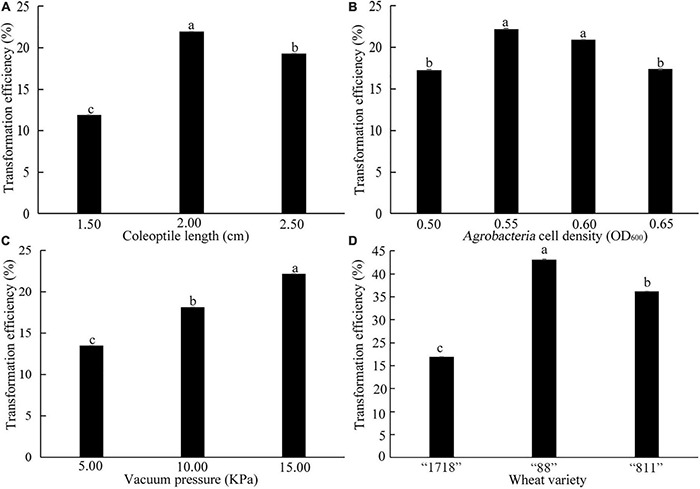
Effects of coleoptile length **(A)**, OD_600_ value of *Agrobacteria* cell density **(B)**, vacuum pressure **(C)**, and wheat varieties **(D)** on transformation efficiency. The three common wheat varieties are “1718,” “88,” and “811.” Bars indicate means with SDs (*n* = 3). Values with different letters are significantly different at *p* < 0.05.

A total of 120 seedlings of common wheat “1718” with coleoptile lengths of 1.50, 2.00, and 2.50 cm were selected. It was found that the most suitable coleoptile length was 2.00 cm, and the transformation efficiency was 21.60% (Figure 8A). When the coleoptile length was 1.50 cm, transformation efficiency was only 11.90%. When the OD value of *Agrobacteria* cell density was 0.55 and 0.60, there was no significant difference in transformation efficiency, which was significantly higher than these of 0.50 and 0.65 OD values (Figure 8B). Therefore, transformation with an *Agrobacteria* cell density OD value of 0.55–0.60 was suitable for common wheat. Transformation efficiency was the highest under a vacuum pressure of 15 KPa and reached 22.16% (Figure 8C). With vacuum pressure falling, transformation efficiency decreased. Therefore, a coleoptile length of 2 cm, *Agrobacteria* cell density of 0.55–0.60 OD_600_, and 15 KPa vacuum pressure were the best for the genetic transformation of common wheat “1718.”

Three common varieties “88,” “811,” and “1718” were transformed by the above transformation system. Their transformation efficiency was 43.15%, 36.18% and 21.98%, respectively (Figure 8D). This result indicated that the coleoptile transformation was robust and reproducible in common wheat.

### Overexpression of *TaLEA2-1* Enhanced Salt Tolerance in Wheat

**FIGURE 9:**
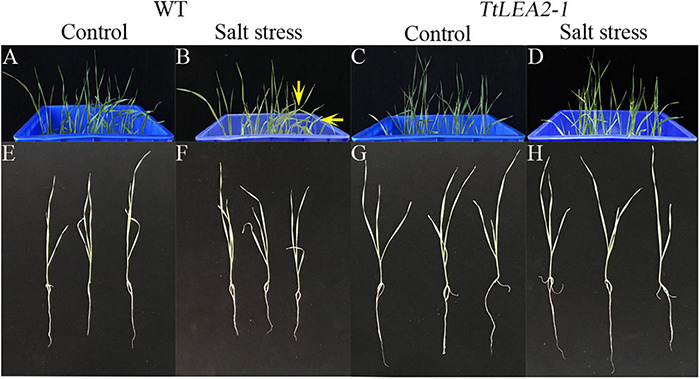
Effects of salt stress treatment on wheat “1718” with *TtLEA2-1* overexpressed**. (A,B)** Control plants of wild-type (WT) grown in a culture box under normal conditions, respectively. Arrows indicate severe wilt. **(C,D)** Transgenic plants grown in a culture box under normal and salt stress conditions, respectively. **(E,F)** and salt stress. **(G,H)** WT whole plants under normal and salt stress conditions, respectively. Images were captured 24 h after salt stress.

Under 250 mM NaCl stress, the leaves of transgenic wheat “1718” were less severely wilted than those of the wild type (WT) plants (Figures 9A–D). In addition, transgenic wheat “1718” displayed greater height and stronger roots than the WT plants, indicating that the *TaLEA2-1* gene conferred salt tolerance in transgenic wheat “1718” (Figures 9E–H).

**FIGURE 10:**
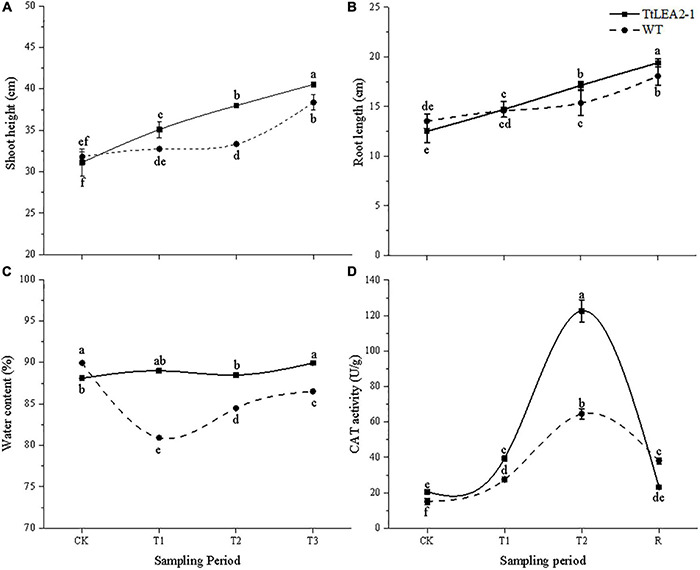
Effect of salt stress and recovery on growth **(A,B)**, plant water content **(C)** and CAT activity **(D)** of transgenic wheat. CK, T1, T2, and R1 represent the control, 5 h after salt stress, 24 h after salt stress, and 1 h after recovery, respectively. Bars indicate means with SDs (*n* = 3). Values with different letters are significantly different at *p* < 0.05.

Under normal conditions, there was no significant difference in root length or seedling height between WT and overexpression plants (Figures 10A,B). The root length and seedling height of overexpression lines were significantly higher than those of WT plants at the T2 (24 h after salt stress) and R1 (1 h after recovery) stages. Overexpression lines showed no significant changes in plant water content under salt stresses. However, plant water content of the WT fell significantly at the T1 (5 h after salt stress), T2, and R1 stages (Figure 10C). These results showed that salt treatment had a greater inhibition effect on WT lines than on overexpression lines, and on roots than on seedlings.

The CAT activity of overexpression lines was higher than that of WT plants under normal and salt stress conditions. Overexpression lines were more sensitive to salt stress than WT plants. The CAT activity of overexpression lines reached a peak at the T2 stage, which was 1.90-fold that of the WT plants. After recovery, the CAT activity of overexpression lines dropped rapidly (Figure 10D).

## Discussion

Salt stress is one of the major abiotic stresses affecting different crops and their yields worldwide (Bhagi et al., 2013). *LEAs* are genes that are closely related to abiotic stress tolerance, such as high salinity stress, drought, and low temperature (Rodriguez-Salazar et al., 2017). LEA protein is highly hydrophilic, which can protect cytoplasmic components and membrane stability under stresses, and prevent further damage to cell proteins (Hand et al., 2011; Hincha and Thalhammer, 2012; Arumingtyas et al., 2013; Furuki and Sakurai, 2016). Eight wheat *TaLEA* genes representing each group were introduced into *E. coli* and yeast to investigate their protective function under heat and salt stress. *TaLEAs* enhanced the tolerance of *E. coli* and yeast to salt and heat, indicating that these proteins have protective functions in host cells under stress conditions (Liu et al., 2019). *JcLEA* from *Jatropha curcas* confers a high level of tolerance to dehydration and salinity in *Arabidopsis thaliana* (Liang et al., 2013). In this study, we cloned a novel *TtLEA2-1* gene from salt-tolerant *Tritipyrum* “Y1805” under salt stress. TtLEA2-1 is a hydrophilic protein with good stability, and rich in aspartic acid and serine, which is consistent with the previous description of LEA proteins (Goyal et al., 2005). Molecular modeling reveals that LEA proteins from wheat have a conserved N-terminal domain, α-helix, and β-chain involved in DNA binding activity (Goyal et al., 2005; Sharma et al., 2021). Here, a good-quality model showed that both N-terminal and C-terminal regions had an α + β-folding chain. The model had the highest number of residues (74.00%) within the favored regions of the Ramachandran plot, making it a reliable 3D protein structure for TtLEA2-1. Meanwhile, the TtLEA2-1 structure was stable by RMSD analysis. This model could pave the way for further functional and structural research of TtLEA2-1.

Late embryogenesis-abundant proteins of common wheat have been classified into eight groups based on sequence similarity and conserved domains (Liu et al., 2019). TtLEA2-1 contains an LEA-2 domain based on the comparison of DNA and amino acid sequences. Moreover, phylogenetic analysis of wheat LEA proteins confirmed that TtLEA2-1 is an LEA-2 protein. Among different groups of LEA proteins, group 2 LEA proteins are biosynthesized in response to salt stress (Allagulova et al., 2003). Group 2 LEAs confer transgenic rice (Ganguly et al., 2012), tobacco (Ju et al., 2021), and wheat (Sharma et al., 2021) with stress tolerance. Here, *TtLEA2-1* might originate from the E genome of *Th. elongatum* according to the transcriptome data. Phylogenetic analysis of LEA proteins from various species also showed that the gene had a close genetic relationship with *Th. elongatum*, indicating that *TtLEA2-1* should originate from the E genome of *Th. elongatum*.

The expression of LEA proteins can be induced by salt stress, drought, and abscisic acid, and is tissue-specific (Zan et al., 2020). Here, the relative expression level of the *TtLEA2-1* gene was the highest in the roots of “Y1805” under salt stress, next was stems, and then leaves (Figure 4A). It was found that the root system was damaged directly and seriously in a high salt solution. In addition, the *TtLEA2-1* expression level in the roots was significantly higher than in the control under salt stress. However, it dropped rapidly to the control level after recovery (Figure 4B). These results were consistent with our transcriptome data and previous report (Zan et al., 2020). Therefore, *TtLEA2-1* was expressed highly and sensitively in the roots to adapt to salt stress.

Although different LEA proteins have different functions, all LEA proteins are widely involved in abiotic stress tolerance in plants (Dhar et al., 2019). GO analysis in *Brachypodium distachyon* demonstrated that LEAs are associated with in “response stimulus,” “developmental process,” “multicellular organismal process,” “multi-organism process,” “immune system process,” and “signaling” ontologies (Ma et al., 2020). LEA genes participate in the protective response of plants to abiotic stresses by reducing reactive oxygen species (Liang et al., 2019; Wang et al., 2021). The *LEA* (*DEHYDRIN3*) gene in grapevine callus is involved in osmotic regulation (Xu et al., 2020). In this work, TtLEA2-1 interacting-proteins were enriched in “peroxisome,” “environmental information processing,” “carbohydrate metabolism,” and “glutamine biosynthetic process” categories, in agreement with previous studies (Liang et al., 2019; Ma et al., 2020; Xu et al., 2020; Wang et al., 2021). Moreover, the translation-related 40S ribosomal protein SA was the central node in PPI analysis, suggesting that it contributes to an efficient reorganization of protein-biosynthesizing machinery in “Y1805” cells (Wang et al., 2013; Saez-Vasquez and Delseny, 2019). *MsLEA-D34* was cloned from alfalfa (*Medicago sativa* L.). An ABA-responsive element (ABRE)-binding transcription factor directly binds to the RY element in the *MsLEA-D34* promoter and activates its expression (Lv et al., 2021). Expression levels of several genes such as *ABF3*, *ABI5*, *NCED5*, and *NCED9* are markedly higher in Arabidopsis plants heterologously expressing *MsLEA4-4* compared to levels in the WT under osmotic stress (Jia et al., 2020). Here, novel TtLEA2-1 interacting proteins related to brassinosteroid signaling, ethylene signaling, and “metabolism of cofactors and vitamins” were found, which may contribute to the salt-tolerance of “Y1805” (Supplementary Table 3).

Despite recent advances in wheat genomic resources, wheat remains one of the most challenging major cereals to genetically transform (Hayta et al., 2019). Biolistic particles (Wei et al., 2016) and *Agrobacterium*-mediated transformation (Cheng et al., 1997) are traditionally applied for genetic transformation in wheat. Anthers, microspores, immature embryos, embryonic calli, and inflorescences are often used as the explants for wheat genetic transformation. The application of these transformed tissues is greatly limited due to various obstacles (such as poor transformation efficiency, somatic variations, albino seedlings, and genotype-dependence) during regeneration in tissue culture (Ghosh et al., 2021). Cells in L1 and L2 layers at the summit of the apex are mitotically active and contribute to the developing shoot and floral structures (Simmonds, 1997). Coleoptile apical meristems have strong meristematic ability and vitality in plants. In this work, the coleoptile tips of common wheat were cut to expose the meristem and then infected with *Agrobacteria* containing the target gene. A 2 cm coleoptile length, *Agrobacteria* cell density of 0.55–0.60 (OD_600 nm_), and 15 KPa vacuum pressure were the best parameters for the transformation of common wheat “1718.” Three common wheat varieties (“88,” “811,” and “1718”) were transformed with this transformation system. It was found that the transformation efficiency was up to 43.15% (Figure 8D). Therefore, this transformation system was robust and reproducible in wheat.

The difficulty of genetic transformation is the bottleneck restricting the development of gene engineering, genetics, breeding, and molecular biology in wheat. The coleoptile transformation of wheat does not depend on tissue culture, which avoids the influence of various factors (such as pollution, vitrification, browning, somaclonal variations, and albino seedlings) that occur during regeneration in tissue culture. An efficient wheat transformation system has now been established, providing the required platform for wheat genetics and breeding. To date, our protocol has been used in our laboratory to introduce more than 20 constructs into wheat. Therefore, this method was simple, time-saving, economical, and genotype-independent in wheat transformation. In addition, this method could be applied to other crop species, especially graminaceous crops (e.g., barley, oats, rice, and corn). Furthermore, this protocol has been applied in the transformation of dicotyledonous species, such as hot peppers, with slight modification in our lab.

## Conclusion

We screened a *Tritipyrum* “Y1805”-specific *LEA* gene because it was expressed highly and sensitively under salt stress using transcriptome analysis. A novel *TtLEA2-1* gene belonging to group 2 was cloned from salt-tolerant “Y1805.” *TtLEA2-1* contains a 453 bp open reading frame encoding a 151-amino-acid protein. *TtLEA2-1* showed maximum homology with *Th. elongatum* by phylogenetic analysis, indicating that *TtLEA2-1* originates from *Th. elongatum*. It is expressed mainly in the roots to adapt to salt stress. The *TtLEA2-1* gene was located in the whole cell. Overall, 48 candidate proteins that are thought to interact with TtLEA2-1 were screened and confirmed by yeast two-hybrid analysis. These interacting proteins were mainly enriched in “genetic information processing,” “environmental information processing,” “glycan biosynthesis and metabolism,” and “carbohydrate metabolism” pathways. The translation-related 40S ribosomal protein SA was the central node by PPI analysis, suggesting that it might play a key role in the salt-stress response of “Y1805.” An efficient wheat transformation system has been established. A coleoptile length of 2 cm, an *Agrobacteria* cell density of 0.55–0.60 OD_600_, and 15 KPa vacuum pressure were ideal for common wheat transformation, with an efficiency of up to 43.15%. *TaLEA2-1* conferred enhanced salt tolerance in transgenic wheat and may be a valuable gene for crop genetic modification.

## Data Availability Statement

The datasets presented in this study can be found in online repositories. The names of the repository/repositories and accession number(s) can be found below: NCBI; PRJNA769794.

## Author Contributions

ZF, GG, and SZ conceived and designed the experiments. ZY, YM, YW, and LS performed the experiments. ZY, YW, and FH analyzed the data. JZ, GG, and QZ contributed reagents, materials, and analysis tools. ZY, GG, and SZ wrote the manuscript. All authors have read and approved the final manuscript.

## Conflict of Interest

The authors declare that the research was conducted in the absence of any commercial or financial relationships that could be construed as a potential conflict of interest.

## Publisher’s Note

All claims expressed in this article are solely those of the authors and do not necessarily represent those of their affiliated organizations, or those of the publisher, the editors and the reviewers. Any product that may be evaluated in this article, or claim that may be made by its manufacturer, is not guaranteed or endorsed by the publisher.

## Footnotes

1
http://www.wheatgenome.org/

2
https://ngdc.cncb.ac.cn/gwh/

3
https://web.expasy.org/protscale/

4
http://www.cbs.dtu.dk/services/NetPhos/

5
http://www.cbs.dtu.dk/services/CPHmodels/

6
http://pfam.xfam.org

7
https://phytozome.jgi.doe.gov/pz/portal.html

8
http://plants.ensembl.org/Triticum_aestivum/Info/Index

9
https://salilab.org/modeller/

10
https://saves.mbi.ucla.edu/

11
http://www.gromacs.org/

12
https://saves.mbi.ucla.edu/

## Supplementary Material

Supplementary Figure 1 Alignment of *TtLEA2-1* from *Tritipyrum* “Y1805” and *Tel3E01G270600* from *Th. elongatum*. Gaps indicate different bases.

Supplementary Figure 2 TtLEA2-1 is a non-toxic and non-self-activating protein. Both of SD/–Leu/–Trp (DDO) plate and SD/–Ade/–His/–Leu/–Trp (QDO) plate have aseptic spots, indicating that the TtLEA2-1 protein was toxic. DDO and QDO plates have plaques, indicating that the TtLEA2-1 protein had self-activation activity. DDO have white plaques and QDO have sterile plaques, indicating that the TtLEA2-1 protein had no self-activating activity.

## References

[B1] Allagulova C. R. Gimalov F. R. Shakirova F. M. Vakhitov V. A. (2003). The plant dehydrins: structure and putative functions. *Biochemistry* 68 945–951. 10.1023/A:1026077825584 14606934

[B2] Amara I. Zaidi I. Masmoudi K. Ludevid D. Pagès M. Goday A. (2014). Insights into late embryogenesis abundant (LEA) proteins in plants: from structure to the functions. *Am. J. Plant Sci.* 5 3440–3455. 10.4236/ajps.2014.522360

[B3] Anders S. Huber W. (2010). Differential expression analysis for sequence count data. *Genome Biol.* 11:R106. 10.1038/npre.2010.4282.1 PMC3218662 20979621

[B4] Arumingtyas E. L. Savitri E. S. Purwoningrahayu R. D. (2013). Protein profiles and dehydrin accumulation in some soybean varieties (*Glycine max* L. merr) in drought stress conditions. *Am. J. Plant Sci.* 4 134–141. 10.4236/ajps.2013.41018

[B5] Bates L. S. Waldren R. P. Teare I. D. (1973). Rapid determination of free proline for water-stress studies. *Plant Soil* 39 205–207. 10.1007/BF00018060

[B6] Battaglia M. Covarrubias A. A. (2013). Late embryogenesis abundant (LEA) proteins in legumes. *Front. Plant Sci.* 4:190. 10.3389/fpls.2013.00190 23805145 PMC3691520

[B7] Bhagi P. Zhawar V. K. Gupta A. K. (2013). Antioxidant response and *Lea* genes expression under salt stress and combined salt plus water stress in two wheat cultivars contrasting in drought tolerance. *Ind. J. Exp. Biol.* 51 746–757. 10.1109/JBHI.2013.2261819 24377135

[B8] Bhalla P. L. (2006). Genetic engineering of wheat–current challenges and opportunities. *Trends Biotechnol*. 24 305–311. 10.1016/j.tibtech.2006.04.008 16682090

[B9] Borrill P. Harrington S. A. Uauy C. (2019). Applying the latest advances in genomics and phenomics for trait discovery in polyploid wheat. *Plant J.* 97 56–72. 10.1111/tpj.14150 30407665 PMC6378701

[B10] Cheng M. Fry J. E. Pang S. Zhou H. Hironaka C. M. Duncan D. R. (1997). Genetic transformation of wheat mediated by *Agrobacterium tumefaciens*. *Plant Physiol*. 115 971–980. 10.1104/pp.115.3.971 12223854 PMC158560

[B11] Cheng Z. Zhang X. Yao W. Zhao K. Liu L. Fan G. (2021). Genome-wide search and structural and functional analyses for late embryogenesis-abundant (LEA) gene family in poplar. *BMC Plant Biol.* 21:110. 10.1186/s12870-021-02872-3 33627082 PMC7903804

[B12] Choa H. W. Shin S. Song K. D. Park J. W. Choi J. Y. Lee H. K. (2015). Molecular characterization and expression analysis of adrenergic receptor beta 2 (*ADRB2*) gene before and after exercise in the horse. *Asian Australas*. *J*. *Anim*. *Sci*. 28 686–690. 10.5713/ajas.14.0575 25924960 PMC4412999

[B13] Cuevas-Velazquez C. L. Rendon-Luna D. F. Covarrubias A. A. (2014). Dissecting the cryoprotection mechanisms for dehydrins. *Front. Plant Sci*. 5:583. 10.3389/fpls.2014.00583 25400649 PMC4212605

[B14] Debnath M. Pandey M. Bisen P. S. (2011). An omics approach to understand the plant abiotic stress. *Omics* 15 739–762. 10.1089/omi.2010.0146 22122668

[B15] Dhar S. Bhattacharya S. Banerjee A. Ray S. (2019). Evolutionary, gene ontology and physiochemical relationships in LEA proteins of *Oryza sativa indica*: detailed computational sequence-based insight. *Plant Gene* 21:100218. 10.1016/j.plgene.2019.100218

[B16] Eswar N. Webb B. Marti-Renom M. A. Madhusudhan M. S. Eramian D. Shen M. Y. (2006). Comparative protein structure modeling using Modeller. *Curr. Protoc. Bioinform.* 5 1–47. 10.1002/0471250953.bi0506s15 18428767 PMC4186674

[B17] Filho G. A. D. S. Ferreira B. S. Dias J. M. Queiroz K. S. Branco A. T. Bressan-Smith R. E. (2003). Accumulation of SALT protein in rice plants as a response to environmental stresses. *Plant Sci*. 164 623–628. 10.1016/S0168-9452(03)00014-1

[B18] Furuki T. Sakurai M. (2016). Group 3 LEA protein model peptides protect enzymes against desiccation stress. *Biochim. Biophys. Acta* 1864 1237–1243. 10.1016/j.bbapap.2016.04.012 27131872

[B19] Ganguly M. Datta K. Roychoudhury A. Gayen D. Sengupta D. N. Datta S. K. (2012). Overexpression of *Rab16A* gene in indica rice variety for generating enhanced salt tolerance. *Plant Signal. Behav.* 7 502–509. 10.4161/psb.19646 22499169 PMC3419040

[B20] Ghosh A. Igamberdiev A. U. Debnath S. C. (2021). Tissue culture-induced DNA methylation in crop plants: a review. *Mol. Biol. Rep.* 48 823–841. 10.1007/s11033-020-06062-6 33394224

[B21] Goyal K. Walton L. J. Tunnacliffe A. (2005). LEA proteins prevent protein aggregation due to water stress. *Biochem. J.* 388 151–157. 10.1042/BJ20041931 15631617 PMC1186703

[B22] Hand S. C. Menze M. A. Toner M. Boswell L. Moore D. (2011). LEA proteins during water stress: not just for plants anymore. *Annu. Rev. Physiol*. 73 115–134. 10.1146/annurev-physiol-012110-142203 21034219

[B23] Hanin M. Brini F. Ebel C. Toda Y. Takeda S. Masmoudi K. (2011). Plant dehydrins and stress tolerance: versatile proteins for complex mechanisms. *Plant Signal. Behav.* 6 1503–1509. 10.4161/psb.6.10.17088 21897131 PMC3256378

[B24] Hayta S. Smedley M. A. Demir S. U. Blundell R. Hinchliffe A. Atkinson N. (2019). An efficient and reproducible *Agrobacterium*-mediated transformation method for hexaploid wheat (*Triticum aestivum* L.). *Plant Methods* 15:121. 10.1186/s13007-019-0503-z 31673278 PMC6815027

[B25] Hess B. Kutzner C. Van D. S. D. Lindahl E. (2008). GROMACS 4: algorithms for highly efficient, load-balanced, and scalable molecular simulation. *J. Chem. Theory Comput*. 4 435–447. 10.1021/ct700301q 26620784

[B26] Hincha D. K. Thalhammer A. (2012). LEA proteins: IDPs with versatile functions in cellular dehydration tolerance. *Biochem. Soc. Trans.* 40 1000–1003. 10.1042/BST20120109 22988854

[B27] Hirayama T. Shinozaki K. (2010). Research on plant abiotic stress responses in the post-genome era: past, present and future. *Plant J*. 61 1041–1052. 10.1111/j.1365-313x.2010.04124.x 20409277

[B28] Hu T. Z. Zhou N. Fu M. L. Qin J. Huang X. Y. (2016). Characterization of OsLEA1a and its inhibitory effect on the resistance of *E. coli* to diverse abiotic stresses. *Int. J. Biol. Macromol.* 91 1010–1017. 10.1016/j.ijbiomac.2016.06.056 27339321

[B29] Huang L. P. Zhang M. Y. Jia J. Zhao X. X. Huang X. X. Ji E. (2018). An atypical late embryogenesis abundant protein OsLEA5 plays a positive role in ABA-induced antioxidant defense in *Oryza sativa* L. *Plant Cell Physiol*. 59 916–929. 10.1093/pcp/pcy035 29432551

[B30] Jia H. Wang X. Shi Y. Wu X. Wang Y. Liu J. (2020). Overexpression of *Medicago sativa LEA4-4* can improve the salt, drought, and oxidation resistance of transgenic *Arabidopsis*. *PLoS One* 15:e234085. 10.1371/journal.pone.0234085 32497140 PMC7272090

[B31] Ju H. Li D. Li D. Yang X. Liu Y. (2021). Overexpression of ZmDHN11 could enhance transgenic yeast and tobacco tolerance to osmotic stress. *Plant Cell Rep.* 40 1723–1733. 10.1007/s00299-021-02734-0 34142216

[B32] Jyothsnakumari G. Thippeswamy M. Veeranagamallaiah G. Sudhakar C. (2009). Differential expression of LEA proteins in two genotypes of mulberry under salinity. *Biol. Plantarum* 53 145–150. 10.1007/s10535-009-0022-2

[B33] Kumar S. Stecher G. Tamura K. (2016). MEGA7: molecular evolutionary genetics analysis version 7.0 for bigger datasets. *Mol. Biol. Evol*. 33 1870–1874. 10.1093/molbev/msw054 27004904 PMC8210823

[B34] Liang J. Zhou M. Q. Zhou X. Jin Y. J. Xu M. Lin J. (2013). JcLEA, a novel LEA-like protein from *Jatropha curcas*, confers a high level of tolerance to dehydration and salinity in *Arabidopsis thaliana*. *PLoS One* 8:e83056. 10.1371/journal.pone.0083056 24391737 PMC3877014

[B35] Liang Y. Kang K. Gan L. Ning S. B. Xiong J. Y. Song S. Y. (2019). Drought-responsive genes, late embryogenesis abundant group3 (*LEA3*) and vicinal oxygen chelate, function in lipid accumulation in *Brassica napus* and *Arabidopsis* mainly via enhancing photosynthetic efficiency and reducing ROS. *Plant Biotechnol. J.* 17 2123–2142. 10.1111/pbi.13127 30972883 PMC6790364

[B36] Liu H. Xing M. Y. Yang W. B. Mu X. Q. Wang X. Lu F. (2019). Genome-wide identification of and functional insights into the late embryogenesis abundant (*LEA*) gene family in bread wheat (*Triticum aestivum*). *Sci. Rep.* 9:13375. 10.1038/s41598-019-49759-w 31527624 PMC6746774

[B37] Liu Y. Wang L. Jiang S. S. Pan J. W. Cai G. H. Li D. Q. (2014). Group 5 LEA protein, *ZmLEA5C*, enhance tolerance to osmotic and low temperature stresses in transgenic tobacco and yeast. *Plant Physiol. Biochem.* 84 22–31. 10.1016/j.plaphy.2014.08.016 25240107

[B38] Lovell S. C. Davis I. W. Arendall W. B. 3rd. Bakker P. I. W. Word J. M. Prisant M. G. (2003). Structure validation by Cα geometry: Φ, Ψ and Cβ deviation. *Proteins: Struct. Funct. Genet.* 50 437–450. 10.1002/prot.10286 12557186

[B39] Luo D. Hou X. M. Zhang Y. M. Meng Y. C. Zhang H. F. Liu S. Y. (2019). *CaDHN5*, a dehydrin gene from pepper, plays an important role in salt and osmotic stress responses. *Int. J. Mol. Sci.* 20:1989. 10.3390/ijms20081989 31018553 PMC6514665

[B40] Lv A. M. Su L. T. Wen W. W. Fan N. N. Zhou P. An Y. (2021). Analysis of the function of the alfalfa *Mslea-D34* gene in abiotic stress responses and flowering time. *Plant Cell Physiol.* 62 28–42. 10.1093/pcp/pcaa121 32976554

[B41] Ma L. T. Zhu T. Wang H. R. Zhou H. Shao L. L. Ding Q. (2020). Genome-wide identification, phylogenetic analysis and expression profiling of the late embryogenesis-abundant (LEA) gene family in *Brachypodium distachyon*. *Funct. Plant Biol.* 48 386–401. 10.1071/FP20143 33278911

[B42] Margiotta B. Colaprico G. Urbano M. Veronico G. Tommasi F. Tomaselli V. (2020). Halophile wheatgrass *Thinopyrum elongatum* (Host) D.R. Dewey (*poaceae*) in three Apulian coastal wetlands: vegetation survey and genetic diversity. *Plant Biosyst.* 10 1–31. 10.1080/11263504.2020.1829732

[B43] Park S. C. Kim Y. H. Jeong J. C. Kim C. Y. Lee H. S. Bang J. W. (2011). Sweet potato late embryogenesis abundant 14 (*IbLEA14*) gene influences lignification and increases osmotic- and salt stress-tolerance of transgenic calli. *Planta* 233 621–634. 10.1007/s00425-010-1326-3 21136074

[B44] Peng Z. Wang Y. Q. Geng G. D. Yang R. Yang Z. F. Yang C. M. (2022). Comparative analysis of physiological, enzymatic, and transcriptomic responses revealed mechanisms of salt tolerance and recovery in *Tritipyrum*. *Front. Plant Sci*. 12:800081. 10.3389/fpls.2021.800081 35069658 PMC8766340

[B45] Qiao L. X. Jiang P. P. Tang Y. Y. Pan L. L. Ji H. C. Zhou W. J. (2021). Characterization of *AhLea-3* and its enhancement of salt tolerance in transgenic peanut plants. *Electro. J. Biotechnol.* 49 42–49. 10.1016/j.ejbt.2020.10.006

[B46] Rahman L. N. Smith G. S. T. Bamm V. V. Voyer-Grant J. A. M. Moffatt B. A. Dutcher J. R. (2011). Phosphorylation of *Thellungiella salsuginea* dehydrins TsDHN-1 and TsDHN-2 facilitates cation-induced conformational changes and actin assembly. *Biochemistry* 50 9587–9604. 10.1021/bi201205m 21970344

[B47] Risacher T. Craze M. Bowden S. Paul W. Barsby T. (2009). Highly efficient *Agrobacterium*-mediated transformation of wheat via in planta inoculation. *Methods Mol. Biol*. 478 115–124. 10.1007/978-1-59745-379-0_7 19009442

[B48] Rodriguez-Salazar J. Moreno S. Espín G. (2017). LEA proteins are involved in cyst desiccation resistance and other abiotic stresses in *Azotobacter vinelandii*. *Cell Stress Chaperones* 22 397–408. 10.1007/s12192-017-0781-1 28258486 PMC5425371

[B49] Rorat T. (2006). Plant dehydrins: tissue location, structure and function. *Cell. Mol. Biol. Lett*. 11 536–556. 10.2478/s11658-006-0044-0 16983453 PMC6275985

[B50] Rosales R. Romero I. Escribano M. I. Merodio C. Sanchez-Ballesta M. T. (2014). The crucial role of Φ- and K-segments in the in vitro functionality of Vitis vinifera dehydrin DHN1a. *Phytochemistry* 108 17–25. 10.1016/j.phytochem.2014.10.006 25457499

[B51] Saez-Vasquez J. Delseny M. (2019). Ribosome biogenesis in plants: from functional 45S ribosomal DNA organization to ribosome assembly factors. *Plant Cell* 31 1945–1967. 10.1105/tpc.18.00874 31239391 PMC6751116

[B52] Sharma A. D. Rakhra G. Vyas D. (2021). Expression analysis and molecular modelling of hydrophilin LEA-2-like gene from wheat. *Plant Cell Rep.* 40 1723–1733. 10.1007/s40502-021-00615-y34142216

[B53] Simmonds J. A. (1997). Mitotic activity in wheat shoot apical meristems: effect of dissection to expose the apical dome. *Plant Sci.* 130 217–225. 10.1016/s0168-9452(97)00206-9

[B54] Thalhammer A. Hincha D. K. (2013). “The function and evolution of closely related COR/LEA (cold-regulated/late embryogenesis abundant) proteins in *Arabidopsis thaliana*,” in *Plant and Microbe Adaptations to Cold in a Changing World*, eds Imai R. Yoshida M. Matsumoto N. (Boston, MA: Springer), 89–105.

[B55] Tolleter D. Hincha D. K. Macherel D. (2010). A mitochondrial late embryogenesis abundant protein stabilizes model membranes in the dry state. *Biochim. Biophys. Acta* 1798 1926–1933. 10.1016/j.bbamem.2010.06.029 20637181

[B56] Trapnell C. Williams B. A. Pertea G. Mortazavi A. Kwan G. Baren M. J. V. (2010). Transcript assembly and abundance estimation from RNA-Seq reveals thousands of new transcripts and switching among isoforms. *Nat. Biotechnol.* 28 511–515. 10.1038/nbt.1621 20436464 PMC3146043

[B57] Wang J. Lan P. Gao H. Zheng L. Li W. Schmidt W. (2013). Expression changes of ribosomal proteins in phosphate-and iron-deficient Arabidopsis roots predict stress-specific alterations in ribosome composition. *BMC Genomics* 14:783. 10.1186/1471-2164-14-783 24225185 PMC3830539

[B58] Wang K. Riaz B. Ye X. G. (2018). Wheat genome editing expedited by efficient transformation techniques: progress and perspectives. *Crop J.* 6 22–31. 10.1016/j.cj.2017.09.009

[B59] Wang Z. D. Zhang Q. Qin J. Xiao G. S. Zhu S. S. Hu T. Z. (2021). *OsLEA1a* overexpression enhances tolerance to diverse abiotic stresses by inhibiting cell membrane damage and enhancing ROS scavenging capacity in transgenic rice. *Funct. Plant Biol.* 48 860–870. 10.1071/FP20231 33820598

[B60] Wei X. Shen F. Hong Y. Rong W. Du L. Liu X. (2016). The wheat calcium-dependent protein kinase TaCPK7-D positively regulates host resistance to sharp eyespot disease. *Mol. Plant Pathol.* 17 1252–1264. 10.1111/mpp.12360 26720854 PMC6638438

[B61] Wingen L. U. West C. Leverington W. M. Collier S. Orford S. Goram R. (2017). Wheat landrace genome diversity. *Genetics* 205 1657–1676. 10.1534/genetics.116.194688 28213475 PMC5378120

[B62] Xu M. L. Tong Q. Wang Y. Wang Z. M. Xu G. Z. Elias G. K. (2020). Transcriptomic analysis of grapevine *LEA* gene family in response to osmotic and cold stress, and functional analyses of *VamDHN3* gene. *Plant Cell Physiol.* 61 4–27. 10.1093/pcp/pcaa004 31967299 PMC10199170

[B63] Yoo S. D. Cho Y. H. Sheen J. (2007). *Arabidopsis* mesophyll protoplasts: a versatile cell system for transient gene expression analysis. *Nat. Protoc.* 2 1565–1572. 10.1038/nprot.2007.199 17585298

[B64] Yuan W. Y. Tomita M. (2015). *Thinopyrum ponticum* chromatin-integrated wheat genome shows salt-tolerance at germination stage. *Int. J. Mol. Sci.* 16 4512–4517. 10.3390/ijms16034512 25809604 PMC4394433

[B65] Zan T. Li L. Q. Li J. T. Zhang L. Li X. J. (2020). Genome-wide identification and characterization of late embryogenesis abundant protein-encoding gene family in wheat: evolution and expression profiles during development and stress. *Gene* 736:144422. 10.1016/j.gene.2020.144422 32007584

