# Supplementary figures and images for: Characterization of a Novel TtLEA2 Gene From Tritipyrum and Its Transformation in Wheat to Enhance Salt Tolerance
Source: Front Plant Sci. 2022 Apr 4;13:830848. doi: 10.3389/fpls.2022.830848 (PMC9014267; doi:10.3389/fpls.2022.830848)

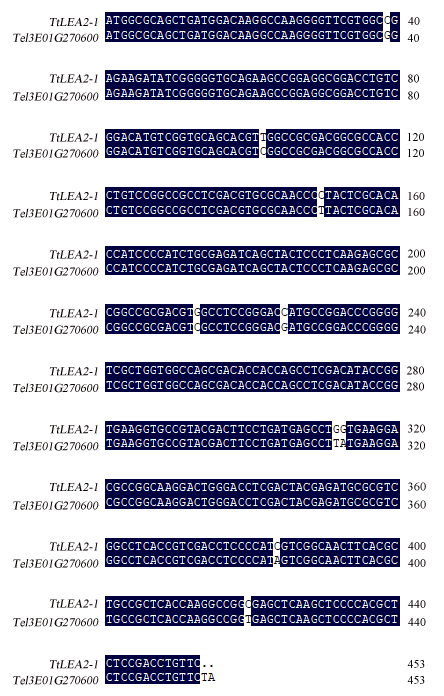

Supplement: Supplementary Figure 1 — Alignment of TtLEA2-1 from Tritipyrum “Y1805” and Tel3E01G270600 from Th. elongatum. Gaps indicate different bases. [file Image_1.TIF]

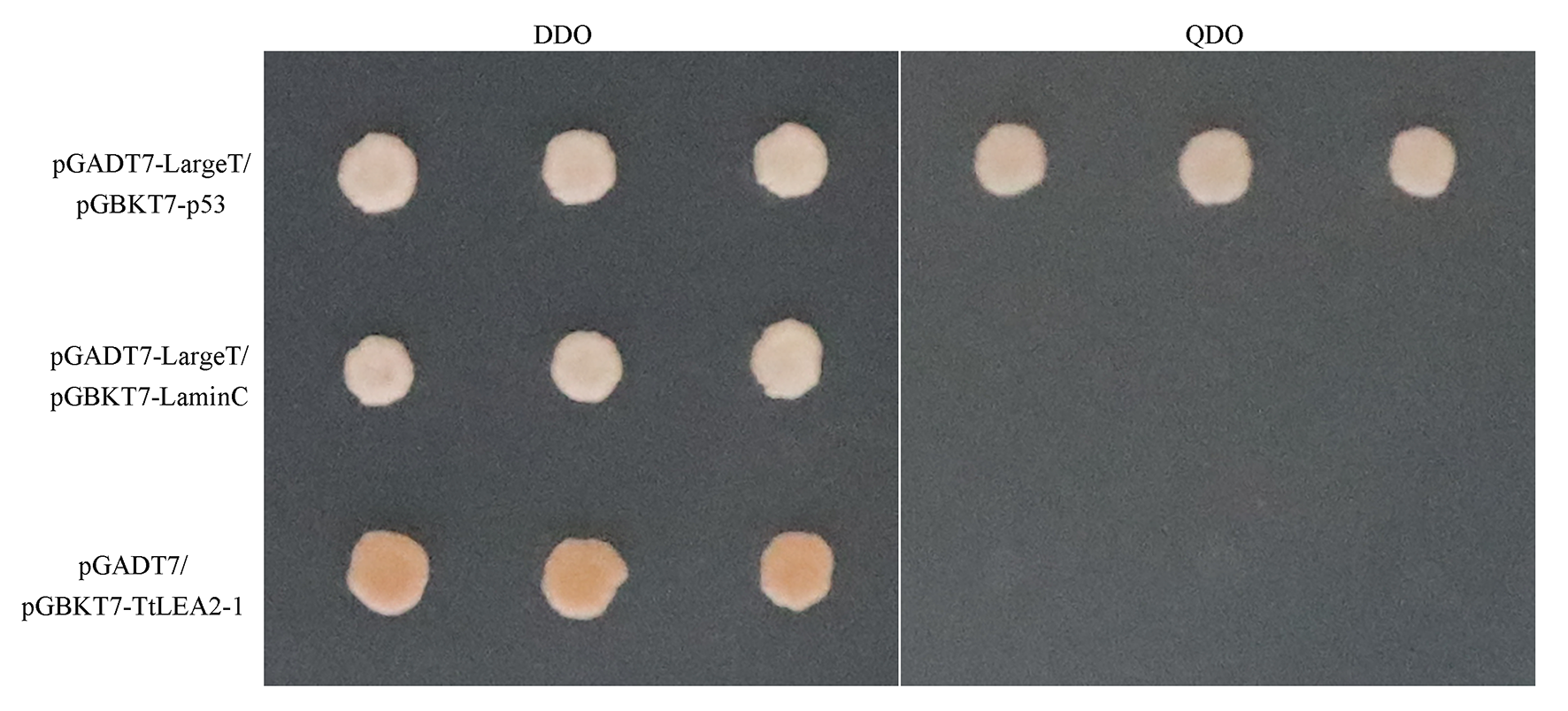

Supplement: Supplementary Figure 2 — TtLEA2-1 is a non-toxic and non-self-activating protein. Both of SD/–Leu/–Trp (DDO) plate and SD/–Ade/–His/–Leu/–Trp (QDO) plate have aseptic spots, indicating that the TtLEA2-1 protein was toxic. DDO and QDO plates have plaques, indicating that the TtLEA2-1 protein had self-activation activity. DDO have white plaques and QDO have sterile plaques, indicating that the TtLEA2-1 protein had no self-activating activity. [file Image_2.TIF]
